# Three Cases of Reversible Agranulocytosis after Treatment with Lamotrigine

**DOI:** 10.4306/pi.2008.5.2.121

**Published:** 2008-06-30

**Authors:** Yong Min Ahn, Kunjong Kim, Yong Sik Kim

**Affiliations:** Department of Psychiatry, and Behavioral Science, Seoul National University College of Medicine, Seoul, Korea.

**Keywords:** Lamotrigine, Agranulocytosis, Bipolar disorder

## Abstract

Several psychotropic drugs, including clozapine, are known to cause agranulocytosis and this may lead to a fatal condition. Lamotrigine is an anticonvulsant that the Food and Drug Administration (FDA) has approved for the depression of bipolar disorder. A few cases of lamotrigine-induced agranulocytosis have been previously reported on, but the pathophysiology and clinical manifestations are not yet known. This case series reports on 3 patients with different medical conditions who experienced agranulocytosis during treatment with lamotrigine. In these cases, the agranulocytosis occurred a few weeks after initiation of lamotrigine and it rapidly disappeared after discontinuation. We also discuss several characteristics of lamotrigine-induced agranulocytosis.

## Introduction

Lamotrigine is ananticonvulsant that the Food and Drug Administration (FDA) has approved for the depression of bipolar disorder. Though skin rashes are the most well known side effect of lamotrigine, blood dyscrasias such as neutropenia, thrombocytopenia and pancytopenia have also been reported.^1^ Furthermore, a few cases of lamotrigine-induced agranulocytosis have been reported,^2^-^5^ but its exact pathophysiology is as yet unknown.^6^ It has been reported that half of the patients with bipolar disorder may take at least 3 psychotropic drugs,^7^ and that the usage of multiple drugs increases the risk of agranulocytosis.^8^ Therefore, it is necessary to be aware of the possibility of agranulocytosis during the treatment of bipolar disorder with lamotrigine.^7^ We report here on 3 cases of agranulocytosis with their different characteristics during treatment with lamotrigine.

## Cases

### Case 1

A 20-year-old man was admitted to a psychiatric ward due to depressive mood of bipolar I disorder. The WBC count and absolute neutrophil count (ANC) at the time of admission were 4,400 cells/mm^3^ and 2240 cells/mm^3^, respectively.

On day 2, treatment with lamotrigine (12.5 mg/day) and amisulpiride was initiated and the lamotrigine was increased to 75 mg/day after 4 weeks with 200 mg of amisulpiride. Fluoxetine was prescribed at week 5, along with increasing the dose of lamotrigine to 100 mg/day by week 6.

**FIGURE 1:**
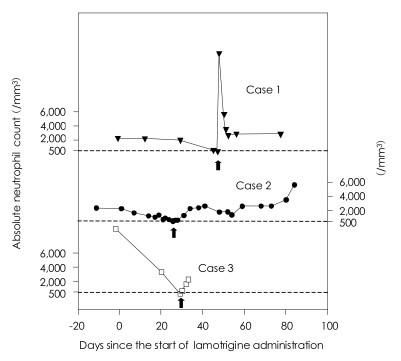
Changes of the absolute neutrophil counts after treatment with lamotrigine.

A CBC at the 45^th^ day after starting the lamotrigine revealed a WBC count of 2,390 cells/mm^3^ and an ANC count of 574 cells/mm^3^. The lamotrigine was discontinued immediately, while the other concurrent drugs were maintained. At the next day, a CBC revealed an ANC count of 380 cells/mm^3^ with agranulocytosis. Although there were no signs of infection, we injected 30 µg of G-CSF and filgrastim, after consulting a hematologist. An ANC count on the 6^th^ day after lamotrigine discontinuation was 3,503 cells/mm^3^ with a normal WBC count (Figure 1).

### Case 2

A 19-year-old woman was treated for bipolar II disorder with quetiapine, paroxetine, trazodone, and valproic acid for about 6 months before admission. During this period, she presented with mild neutropenia (ANC count: 1000-1500 cells/mm^3^), and the cause of this was assumed to be the valproic acid. After the valproic acid was discontinued about 3 months before admission, her ANC count was normalized.

Due to the aggravation of her suicidal ideation, she was admitted and then started on 25 mg of lamotrigine. A CBC done at the time of admission showed a WBC count of 4,900 cells/mm^3^ and an ANC count of 2,328 cells/mm^3^. Neutropenia manifested on day 14 (an ANC count of 1,282 cells/mm^3^), but upon consultation with a hematologist, the lamotrigine prescription was continued upon consideration of her previous history of persistent neutropenia. On day 24, her ANC count was reduced to 720 cells/mm^3^, so on day 26, the dosage of lamotrigine was reduced to 12.5 mg/day. But on day 27 there was an outbreak of agranulocytosis (ANC count: 491 cells/mm^3^), and the lamotrigine was discontinued immediately (concurrent medications: 5 mg of olanzapine, 200 mg of quetiapine and 50 mg of trazodone) (Figure 1). On the 7^th^ day after lamotrigine discontinuation, her ANC count (2,330 cells/mm^3^) was normalized (Figure 1).

Due to her severe suicidal ideation, clozapine was initiated about 33 days later and the lamotrigine was reintroduced approximately 100 days after its discontinuation. Now, about 27 months later, she is on 300 mg of lamotrigine and 262.5 mg of clozapine without any hematologic abnormalities.

### Case 3

A 27-year-old man had been taking risperidone for 6 years for the treatment of bipolar I disorder. He was admitted due to a depressive mood and auditory hallucinations. A CBC done on admission day showed a WBC count of 12,670 cells/mm^3^ and an ANC count of 9,541 cells/mm^3^. The patient was started on lamotrigine (25 mg/day) and this was increased to 100 mg/day 18 days later. On the 20^th^ day, 12.5 mg of clozapine was added and a CBC revealed a WBC count of 6,510 cells/mm^3^ and an ANC count of 3,431 cells/mm^3^.

On the 29^th^ day after lamotrigine initiation (the 9^th^ day after clozapine initiation), a CBC revealed an ANC count of 348 cells/mm^3^ with agranulocytosis (concurrent medication: 4 mg of risperidone and 175 mg of clozapine). The lamotrigine was discontinued immediately. On the 3^rd^ day after discontinuation, the patient's ANC count increased to 2,367 cells/mm^3^ (Figure 1). Afterwards, the risperidone was discontinued, and the dosage of clozapine was increased to 225 mg, and there have not been any hematologic abnormalities since then.

## Discussion

In case 1 and 2, although the other concurrently administered drugs could not be definitely excluded from the possibility of inducing agranulocytosis,^8^ it is relatively easy to consider lamotrigine as the cause of agranulocytosis because the patients had been taking other drugs for over 6 months without any hematologic abnormalities. In case 3, though it was difficult to make the proper clinical judgment, lamotrigine was supposed to be the cause of agranulocytosis because clozapine had been taken only for 9 days, which is too short a time to cause agranulocytosis.^9^

In these 3 cases, agranulocytosis occurred on days 46, 27 and 29 after lamotrigine initiation, respectively, and in the 3 previously reported cases,^2^-^4^ the agranulocytosis also occurred within 8 weeks. It is likely that lamotrigine-induced agranulocytosis has a tendency to occur at the early phase of treatment, which is unlike clozapine that rarely induces agranulocytosis within the first 6 weeks.^9^

In cases 1 and 3, the agranulocytosis occurred 4 days after an increased titration of lamotrigine. In the 3 previously reported cases, the durations between an increased dose of lamotrigine and the hematologic abnormalities were also 3-4 days.^2^,^10^,^11^ For our cases, the doses of lamotrigine at the onset of agranulocytosis were 100 mg, 25 mg and 150 mg, respectively, and in the previous reports, the doses of lamotrigine ranged from 50 mg to 250 mg. These findings may suggest that the increased titration is more important for inducing agranulocytosis rather than the dosage itself.

There was one previous case report in which lamotrigine was restarted after neutropenia.^10^ They reported that the neutropenia reappeared within 2 days when lamotrigine was restarted after 17 days. But in our case 2, lamotrigine was restarted about 100 days after its discontinuation and there have been no other hematologic abnormalities. Like clozapine,^12^ more experience and data should be accumulated to understand these differences.

In our cases, the patients' hematologic abnormalities were normalized within 1 week after lamotrigine discontinuation, which corresponds with the previous case reports. Since fast recovery is unusual for the cases of clozapine-induced agranulocytosis, lamotrigine-induced agranulocytosis may have a different mechanism for inducing agranulocytosis.

To conclude, at the early phase of lamotrigine treatment or after an increased titration of lamotrigine, it is beneficial for the clinician to aware the possibility of an occurrence of agranulocytosis, although it is very rare and this condition was reversible in our cases.
